# LpMab-23-recognizing cancer-type podoplanin is a novel predictor for a poor prognosis of early stage tongue cancer

**DOI:** 10.18632/oncotarget.24986

**Published:** 2018-04-20

**Authors:** Akihiro Miyazaki, Hiromi Nakai, Tomoko Sonoda, Yoshihiko Hirohashi, Mika K. Kaneko, Yukinari Kato, Yoshihiko Sawa, Hiroyoshi Hiratsuka

**Affiliations:** ^1^ Department of Oral Surgery, Sapporo Medical University School of Medicine, Chuo-ku, Sapporo 060-8543, Japan; ^2^ Department of Public Health, Sapporo Medical University School of Medicine, Chuo-ku, Sapporo 060-8556, Japan; ^3^ Department of Pathology, Sapporo Medical University School of Medicine, Chuo-ku, Sapporo 060-8556, Japan; ^4^ Department of Antibody Drug Development, Tohoku University Graduate School of Medicine, Aoba-ku, Sendai, Miyagi 980-8575, Japan; ^5^ New Industry Creation Hatchery Center, Tohoku University, Aoba-ku, Sendai, Miyagi 980-8575, Japan; ^6^ Deparment of Oral Function & Anatomy, Okayama University Graduate School of Medicine, Dentistry, and Pharmaceutical Sciences, Okayama Kita-ku 700-0914, Japan

**Keywords:** LpMab-23, predictor, podoplanin, tongue cancer, antibody

## Abstract

**Purpose:**

We report that the reactivity of a novel monoclonal antibody LpMab-23 for human cancer-type podoplanin (PDPN) is a predictor for a poor prognosis of tongue cancer.

**Patients and Methods:**

The association between LpMab-23-recognizing cancer-type PDPN expression and clinical/pathological features were analyzed on 60 patients with stage I and II tongue cancer treated with transoral resection of the primary tumor.

**Results:**

In the mode of invasion, the LpMab-23-dull/negative cases were significantly larger in cases with low-grade malignancies and without late cervical lymph node metastasis, than in cases with high-grade malignancies and the metastasis. In the high-grade malignant cases, LpMab-23-positive cases were significantly larger than LpMab-23-dull/negative cases. The Kaplan–Meier curves of the five-year metastasis-free survival rate (MFS) were significantly lower in the LpMab-23 positive patients than in LpMab-23 dull/negative patients. The LpMab-23-dull/negative cases showed the highest MFS in all of the clinical/pathological features and particularly, the MFS of the LpMab-23 positive cases decreased to less than 60% in the first year. In the Cox proportional hazard regression models a comparison of the numbers of LpMab-23 dull/negative with positive cases showed the highest hazard ratio with statistical significance in all of the clinical/pathological features.

**Conclusions:**

LpMab-23 positive cases may be considered to present a useful predictor of poor prognosis for early stage tongue cancer.

## INTRODUCTION

In a number of human cancers, a high level of expression of podoplanin (PDPN) limited to the invasion front of the cancer has been reported in squamous cell carcinomas of the upper digestive tract, from the oral cavity to the esophagus [1–5]. The PDPN expression has also been shown in osteosarcoma, mesothelioma, basal cell carcinoma of the skin, follicular dendritic cell tumors, germ cell tumors of the ovary and testis, and hemangioblastoma, and brain tumors such as astrocytomas or glioblastomas [6–13]. It appears that in some cases of tumors, the high expression of PDPN is closely correlated with the severity of clinical deterioration: invasion, metastasis into regional lymph nodes, recurrences, and shorter survival times [14–18]. There are two explanations for the significance of the high expression of PDPN. One is as an induction of the transdifferentiation of epithelial cells into motile mesenchymal cells, a process known as epithelial–mesenchymal transition (EMT) promotion with E-cadherin downregulation [19]; the other is not concerned with EMT [14], but both of two papers refer to a high expression of PDPN as being related to the enhancement of a high metastatic potential. Different from these studies, however, there are also reports that a low expression of PDPN in uterine cervix cancers as correlated with poorer outcomes: lymphatic metastasis and poorer survival rates [20, 21].

Further, a high expression of PDPN has also been observed in cancer-associated fibroblasts (CAFs) of the stroma surrounding tumors [22–24]. There are some reports that the high expression of PDPN in CAFs is implicated in a poorer prognosis of invasive adenocarcinomas of the lung and pancreas [22–24]. Invasive ductal carcinoma of the pancreas has desmoplasia with abundant fibrous connective tissue. The PDPN is a lymphatic vessel marker (clone D2-40; one of the anti-humanPDPN monoclonal antibodies: mAbs) and expression of PDPN by stromal CAFs has been reported to be a prognostic indicator in various types of cancer. The PDPN-expressing CAFs enhances the progression of invasive ductal carcinomas of the pancreas, and a high ratio of PDPN-expressing CAFs is an independent predictor of poor outcomes. In these cases, PDPN may enhance the tumor-promoting effects of CAFs due to increased RhoA activity [19, 25]. The PDPN plays a key role in the cell process elongation by the actin cytoskeleton rearrangement dependent on the binding activity with a cytoplasmic linker protein ezrin via RhoA family signaling. The PDPN up-regulates a Rho-GTPase activity resulting in ezrin phosphorylation and phosphorylated ezrin mediates the connection of PDPN to F-actin. The PDPN induces the formation of membrane-actin structures and promotes tumor cell invasion via plasma membrane extensions [26, 27]. However, there is also a case where colorectal CAFs with high PDPN expression, which is correlated with a better prognosis, was identified in stroma surrounding the tumors in many areas other than at the invasive front, postulating that PDPN expression on the stromal fibroblasts may act as a barrier to tumor cell invasion [28].

From the above, it appears that there are two conflicting hypotheses for the high expression of PDPN in cancer: one leading to a better prognosis and the other suggesting a poorer prognosis. Many sialic acids bind to PDPN and affect the PDPN conformation by a negative charge, repelling other molecules. Therefore, it may be thought that the PDPN conformation is different for different cancer cell types due to the diversity of sialic acid bindings, and the diversity affects the affinities of antibodies to PDPN, causing the opposing diagnosis for the cancer by PDPN immunostaining. A novel mAb LpMab-23 was recently established for human cancer-type PDPN using the cancer-specific mAb (CasMab) technology [29]. Further, the chimeric antibody chLpMab-23 exhibited antitumor activity via antibody-dependent cellular cytotoxicity [30]. The critical epitope of LpMab-23 is Gly54-Leu64, which is common to both cancer and normal cells. Importantly, LpMab-23 reacts with human cancer cells but not with normal cells such as lymphatic endothelial cells. It may be speculated that the cancer-specific reactivity of LpMab-23 could be due to the cancer specific O-linked glycosylation. The cancer-type podoplanin has Thr55 and Ser56 aberrantly *O*-glycosylated by disialyl-core1 (NeuAc-a2-3Galb1-3 (NeuAca2-6) GalNAca1-*O*-Thr) on an extracellular domain [30]. There is also the possibility that the different reactions of LpMab-23 in cancer cells and normal cells may be a cause of the conformational changes of the epitope with the loss of the glycosylation on cancer cells, further studies to determine this are needed. If there are differences in the D2-40 reaction to oral cancer, which has a well-known reactivity to PDPN of both cancer cells and normal cells, and LpMab-23 with a reactivity that is specific to the oral cancer-type PDPN, it may be thought that PDPN conformation is different for different cancers, resulting in the differences in clinical significance for the PDPN expression. This study aims to investigate whether the reactivity of LpMAb-23 is a predictor of a poor prognosis for early stage tongue cancer.

## RESULTS

### Subjects

Tissue blocks from 60 patients from 33 to 92 years of age (male [n=33], female [n=27]) who had undergone partial glossectomy without elective neck dissection (under 66 years of age [n=33]; and above 66 years of age [n=27]) were tested. All cancers were T1 (tumor 2cm or less in the greatest dimension) or T2 (tumor more than 2cm but not more than 4 cm in the greatest dimension) of the T-classification and Grade 1 (well differentiated)(n=29), Grade 2 (moderately differentiated), or Grade 3 (poorly differentiated)(n=31). For the mode of invasion according to the YK classification, 42 subjects belonged to YK-1 (well defined borderline), YK-2 (cords, less marked borderline), or YK-3 (groups of cells, no distinct borderline) while 14 subjects belonged to YK-4C (diffuse invasion, cord-like type invasion) and 4 belonged to YK-4D (diffuse invasion, diffuse type invasion). There were three subjects with local recurrence and 14 subjects with late cervical lymph node metastasis.

### Immunohistochemical analysis for the reaction of D2-40 and LpMab-23

**Figure 1:**
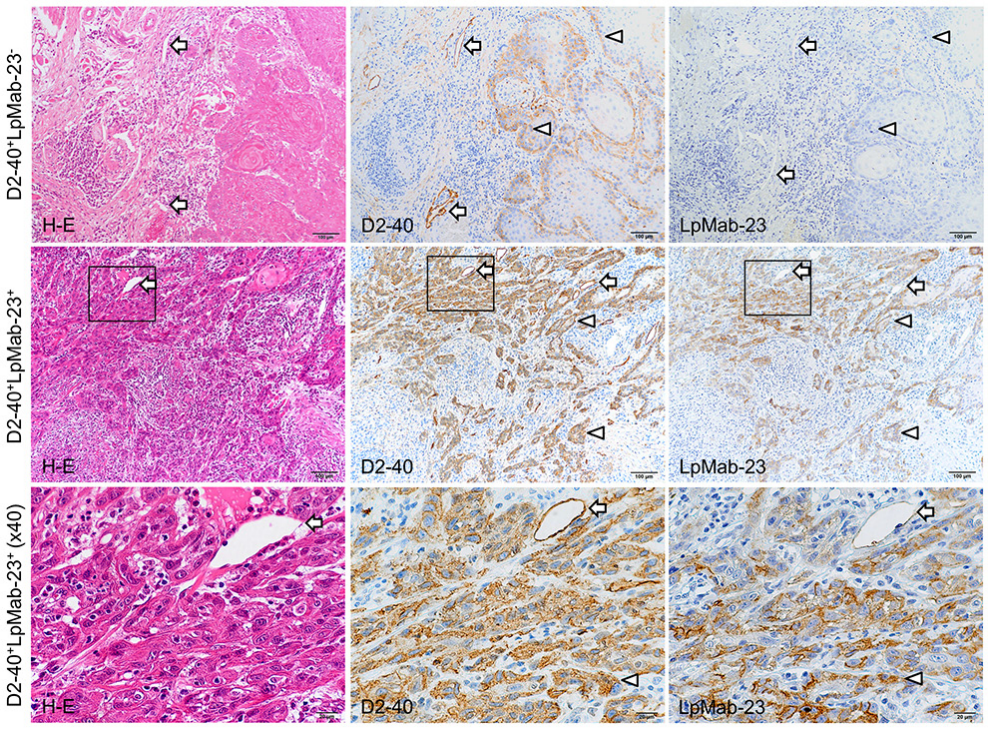
D2-40-positive and LpMab-23-negative/positive early stage tongue squamous cell carcinoma These sections are visualized by DAB staining for reaction products with D2-40 to normal PDPN and with LpMab-23 for the cancer-specific PDPN with Hematoxylin staining of nuclei (blue). The top row shows panels of a D2-40-positive (middle panel) and LpMab-23-negative (right panel) tongue carcinoma (65 year old female, Grade 1, YK-2, no recurrence, no metastasis). Lymphatic vessels are reacted with D2-40 (arrows) and cells of the cancer at the margins are also D2-40 positive on the cell membrane (arrowheads) while in the adjacent section, neither cancer cells nor lymphatic vessels are LpMab-23 negative (arrowheads). Bars: 100 μm. The middle row shows panels of a D2-40 and LpMab-23-double positive tongue carcinoma (57 year old female, Grade 1, YK-4C, no recurrence, with metastasis). Lymphatic vessels are reacted with D2-40 (arrows) but not with LpMab-23. Almost all of the invasive cancer cells are D2-40 positive with many lymphocyte infiltrations similar to LpMab-23 (arrowheads). Bars: 100 μm. The bottom row shows magnified views of the box highlighted in each panel of the middle row. Almost all cancer cells have both membrane and cytoplasm with the diffusion of LpMab-23 reaction products. Bars: 20 μm.

**Figure 2:**
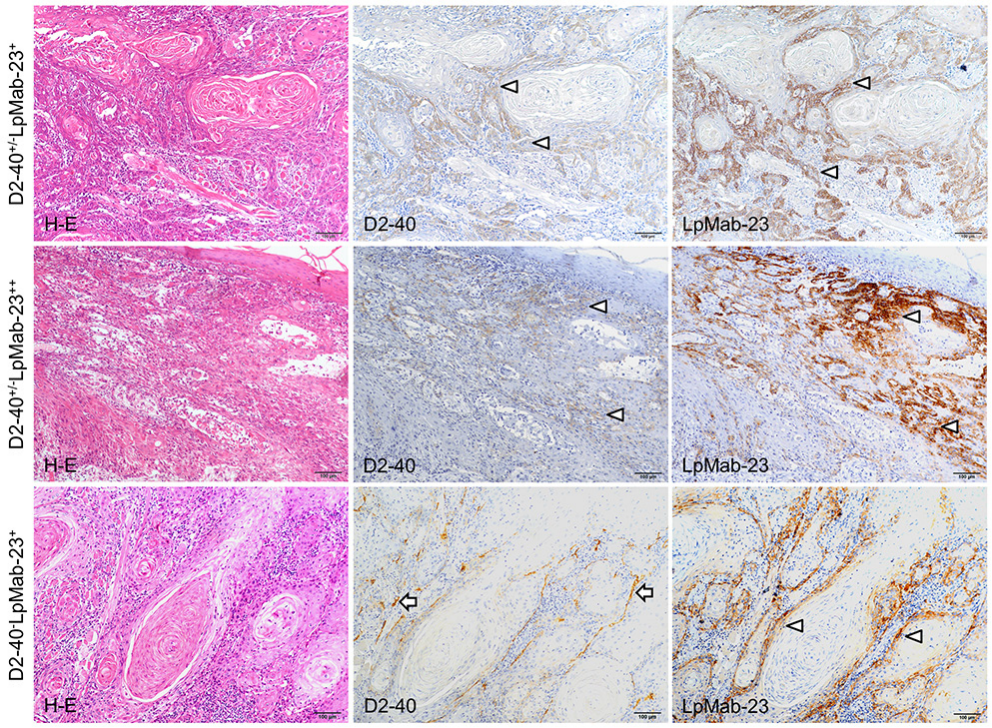
D2-40 dull/negative and LpMab-23 positive early stage tongue squamous cell carcinoma The sections are visualized by DAB staining for reaction products with D2-40 to normal PDPN and LpMab-23 for the cancer-specific PDPN with Hematoxylin staining of nuclei (blue). The top row shows panels of a D2-40 dull (middle) and LpMab-23-positive (right) tongue carcinoma (77 year old female, Grade 2, YK-4C, no recurrence, with metastasis). The cells of the cancer at the margins are weakly reacted with D2-40 (arrows) while in the adjacent section, the reaction with LpMab-23 (arrows) is stronger than that with D2-40. Bars: 100 μm. The middle row shows panels of a D2-40 dull and LpMab-23-positive tongue carcinoma (79 year old female, Grade 1, YK-4C, no recurrence, with metastasis). The invasive cancer cells showed few reactions with D2-40 (arrows) while in the adjacent section the cells showed strong reaction with LpMab-23 (arrows). Bars: 100 μm. The bottom row shows panels of a D2-40 negative and LpMab-23 positive-tongue carcinoma (77 year old female, Grade 2, YK-4C, no recurrence, with lymph node metastasis). The invasive cancer cells show no reactions with D2-40 while there is lymphangiogenesis with much D2-40-positive lymphatic vasculature found among cancer cells (arrowheads). In the adjacent section the cancer cells show a reaction with LpMab-23 (arrows). Bars: 100 μm.

In the study, it was found that it is possible to distinguish 5 cases of combinations of reactions with D2-40 and LpMab-23 to the tongue squamous cell carcinoma: D2-40-positive and LpMab-23-negative; D2-40 and LpMab-23-double positive; D2-40-dull and LpMab-23-positive; D2-40-dull and LpMab-23-strongly positive; D2-40-negative and LpMab-23-positive (Figure 1, Figure 2). Further, reaction of LpMab-23 to cancer cells was found on the cell membrane and cytoplasm (Figure 1). The D2-40 reacted with lymphatic vessels and the marginal cells of cancer, while LpMab-23 did not react with lymphatic vessels in any of the tissue samples tested (Figure 1). There was no cancer tissue without reaction to either D2-40 or LpMab-23. In cancer cells with low malignant potential, D2-40 is positive on the cell membrane but LpMab-23 is more commonly negative (Figure 1). In the D2-40 and LpMab-23-double positive cancer, there were cells which have cytoplasm with reaction products to LpMab-23 (Figure 1). In the D2-40-dull cancers, the reaction to LpMab-23 was more pronounced (Figure 2). It was found that the cancer tissue where almost all cells are LpMab-23 positive has many lymphocyte infiltrations and was composed of cancer cells with cytoplasm showing diffusion of reaction products to LpMab-23 (Figure 2). In cancer tissue with high malignant potential, the cells were commonly D2-40 dull/negative and LpMab-23 positive/strongly positive (Figure 2). Furthermore, active lymphangiogenesis was most commonly found among LpMab-23 positive-cancer cells with no reaction to D2-40 (Figure 2).

### Association between LpMab-23 reactivity and clinical features

There were statistically significant differences in the mode of invasion and late cervical lymph node metastasis among the significant LpMab-23 reactivity vs clinical and pathological parameters (Table 1). In the mode of invasion, significantly more LpMab-23-dull or negative reactions were determined in the YK-1/YK-2/YK-3 group; in the YK-4C and YK-4D groups, the reactions of LpMab-23 were predominantly more positive than dull/negative (P<0.01). In the late cervical lymph node metastasis, significantly more LpMab-23-dull/negative reactions were determined in cancers without metastasis while with metastasis, the reactions of LpMab-23 were predominantly more positive than dull/negative (P<0.01). There were no statistically significant correlations between the LpMab-23 reactivity and gender, age, T-classification, histological grading, and local recurrence (Table 1).

**Table 1:** Relevance between LpMab-23 reactivity and clinical/pathological features

Clinical/pathological features	LpMab-23 reactivity				
	Dull and Negative		Positive		P-value
	No.	%	No.	%	
All patients	29	48.3	31	51.7	NS
Gender					
Male	16	26.7	17	28.3	NS
Female	13	21.7	14	23.3	
Age					
<66	18	30	15	25	NS
≥66	13	21.7	14	23.3	
T-classification					
T1	11	18.3	18	30	NS
T2	18	30	13	21.7	
Histological grading					
Grade 1	15	25	14	23.3	NS
Grade 2, 3	14	23.3	17	28.3	
Mode of invasion					
YK-1, 2, 3	27	45	15	25	P<0.01
YK-4C	2	3.3	12	20	
YK-4D	0	0	4	6.7	
Local recurrence					
No	26	43.3	31	51.7	NS
Yes	3	5	0	0	
Late cervical lymph node metastasis					
No	28	46.7	18	30	P<0.01
Yes	1	1.7	13	21.7	

NS, not significant. Ratio was rounded off to the 1st decimal place.

There were however statistically significant differences in the age, mode of invasion, and LpMab-23 reactivity for the five-year metastasis-free survival rate (MFS)(Table 2). The survival rate was higher in patients under 66 years of age than in patients older than 66 years (p<0.05), and higher in patients with the mode of invasion YK-1/YK-2/YK-3 than in YK-4C and YK-4D patients (p<0.01). Notably, the survival rate was lower in patients with LpMab-23 positive than in patients with dull/negative reactivity (Table 2).

**Table 2:** Relevance between clinical/pathological features including LpMab-23 reactivity and five-year metastasis-free survival rate (MFS)

Clinical/pathological features	Number of patients	MFS (%)	P-value
Gender			
Male	33	84.8	NS
Female	27	66.7	
Age			
<66	33	87.9	P<0.05
≥66	27	63.0	
T-classification			
T1	29	79.3	NS
T2	31	74.2	
Histological grading			
Grade 1	29	86.2	NS
Grade 2, 3	31	67.7	
Mode of invasion			
YK-1, 2, 3	42	88.1	P<0.01
YK-4C	14	57.1	
YK-4D	4	25.0	
LpMab-23 reactivity			
dull and negative	28	96.4	P<0.01
positive	32	59.4	

NS, not significant. Ratio was rounded off to the 1st decimal place.

### Association between LpMab-23 reactivity and prognosis

**Figure 3:**
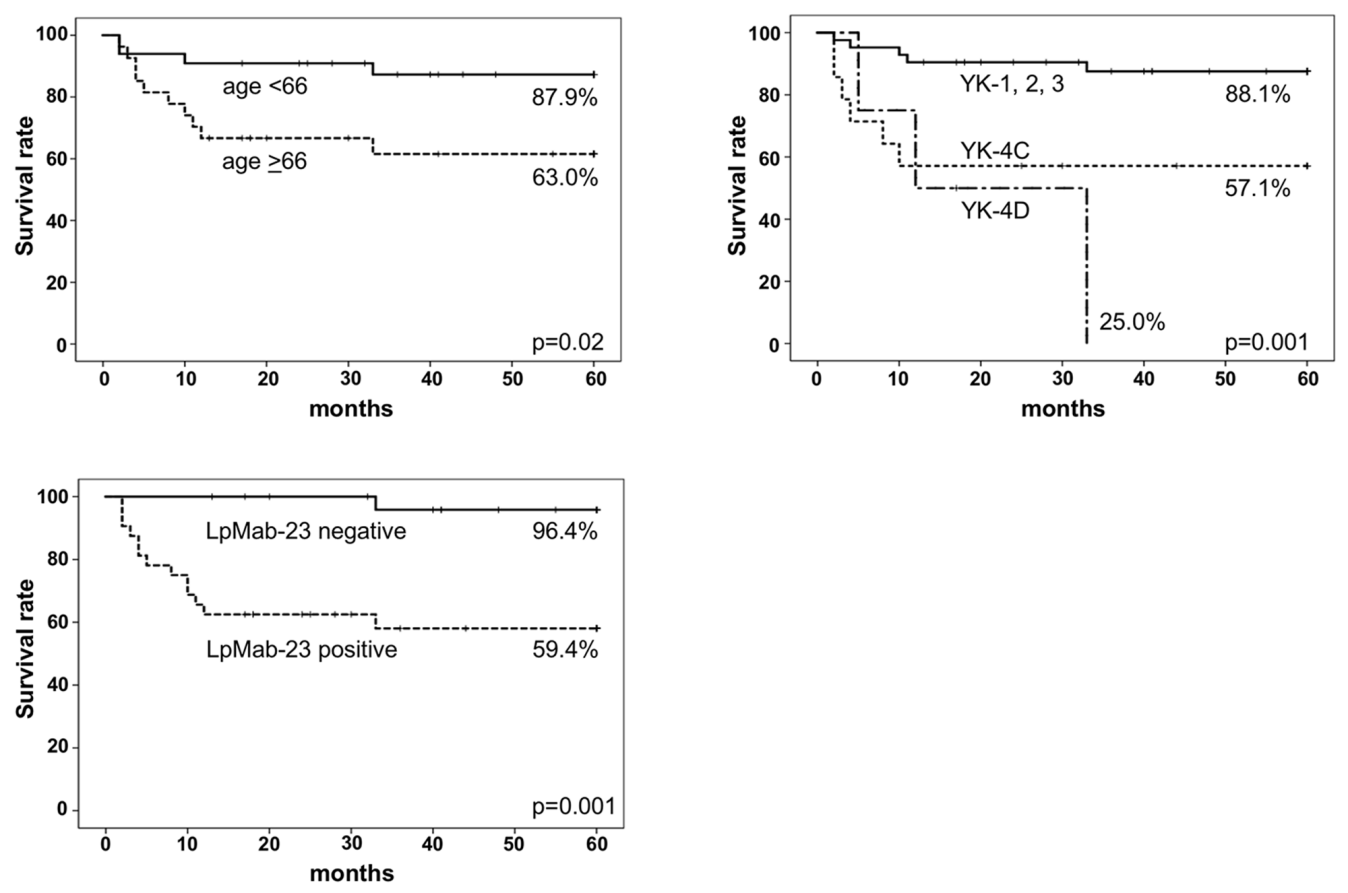
Five-year metastasis-free survival curves (MFS) The Kaplan–Meier curves of MFS were significantly lower in patients aged 66 years or older (n=27) than in the patients with under 66 years of age (n=33)(p=0.02), lower in the patients with mode of invasion YK-4C (n=14) and 4D (n=4) than in the patients with mode of invasion YK-1/2/3 (n=42)(p=0.001), lower in the patients with LpMab-23 positive (n=32) than in the patients with LpMab-23 negative (n=28)(p=0.001). Particularly, the survival rate of patients with LpMab-23 positive decreased to less than 60% in the first year during 60 months of follow-up (long-rank) as well as the patients with the mode of invasion YK-4C and 4D.

The Kaplan–Meier curves of MFS were significantly lower in LpMab-23 positive patients (59.4%), in patients with the mode of invasion YK-4C (57.1%) and YK-4D (25.0%), and patients older than 66 years (63.0%) compared with LpMab-23 negative patients (96.4%), patients with the mode of invasion YK-1/YK-2/YK-3 (88.1%), and patients under 66 years of age (87.9%). The MFS of LpMab-23 positive and YK-4C or 4D patients was lower, decreasing below 60% in the first year of the 60 months of follow-up (long-rank)(Figure 3). In the Cox proportional hazard regression models the LpMab-23 positive vs dull/negative showed the highest hazard ratio and a statistically significantly difference (Table 3). In the univariate analysis, age, mode of invasion, and LpMab-23 reactivity are associated with late cervical lymph node metastasis: significantly different for patients under 66 years vs patients aged 66 years or older, for YK-1/YK-2/YK-3 vs YK-4C vs YK-4D, LpMab-23 dull/negative vs positive (p<0.05). In the multivariate analysis among age, mode of invasion, and LpMab-23 reactivity, LpMab-23 reactivity is only identified as an independent factor; significantly different for LpMab-23 dull/negative vs positive (p<0.05).

**Table 3:** Cox proportional hazard regression models

Clinical/pathological features	Hazard ratio	95% CI	P-value
**Univariate analysis**			
Gender (female vs male)	2.33	0.78 to 6.95	0.13
Age (≥66 vs <66)	3.54	1.11 to 11.32	0.03
T-classification (T1 vs T2)	1.23	0.43 to 3.55	0.70
Histological grading (Grade 1 vs Grade 2, 3)	2.57	0.81 to 8.21	0.11
Mode of invasion (YK-1, 2, 3 vs YK-4C vs YK-4D)	2.92	1.53 to 5.56	<0.01
LpMab-23 (dull/negative vs positive)	14.95	1.95 to 114.65	<0.01
**Multivariate analysis (age, mode of invasion, LpMab-23 reactivity)**			
Age (≥66 vs <66)	2.47	0.76 to 8.07	0.13
Mode of invasion (YK-1, 2, 3 vs YK-4C vs YK-4D)	1.59	0.78 to 3.23	0.20
LpMab-23 reactivity (dull/negative vs positive)	9.33	1.11 to 78.51	0.04

Ratio was rounded off to the 2nd decimal place.

## DISCUSSION

The aim of this study is to investigate the usefulness of LpMab-23, a unique mAb for cancer-type PDPN, as a predictor for a poor prognosis of early stage tongue cancer. The PDPN is a mucin-type highly glycosylated protein identified in podocytes [31], osteocytes of rodent bones [32], type I alveolar cells [33], and lymphatic endothelial cells [3]. The PDPN is also expressed in the choroid plexus [34], mesothelial cells [35], epidermal basal layer cells, tooth germ epithelial cells, salivary gland myoepithelium [36], thymus type I epithelial cells, follicular dendritic cells, and in the central nervous system and perineurium [37]. The D2-40 is a well-known antibody to human PDPN of normal tissue described above while LpMab-23 of which the epitope is Gly54-Leu64 of cancer-type PDPN does not react with PDPN of normal cells [29, 30]. In the immunohistochemical analysis of differences between D2-40 and LpMab-23 in tongue cancer tissue, the reaction to lymphatic vessels was observed in D2-40 but not in LpMab-23 (Figure 1), suggesting that LpMab-23 is specific for cancer-type PDPN. There was no cancer tissue without reaction to either D2-40 or LpMab-23. In LpMab-23-positive tissue, it was observed that cells reacted with LpMab-23 on the cell membrane and cytoplasm (Figure 1, 2). There was diversity in the reactions of D2-40 and LpMab-23 to cancer cells, and there were cases where the reactions to the same subject were similar or different (Figure 1, 2), suggesting that early stage tongue cancer cells produce both normal PDPN and LpMab-23-recognizing cancer-type PDPN at several extents.

The prognosis of oral squamous cell carcinomas depends on cervical lymph node metastasis. It is generally accepted that patients with clinically negative cervical lymph nodes will have a good prognosis, though the prognosis of patients with lymph node metastasis occurring after surgery or radiotherapy of the primary tumor is poor. However, some early stage patients with clinically negative cervical lymph nodes have a poor prognosis because of developing late cervical lymph nodes [38]. Much research has been attempting to identify potential molecular and morphological predictors for late cervical lymph node metastasis, but so far none have made an impact on routine clinical procedures [39]. The YK classification focused on the shape of tumor-cell cords at the tumor-host interface has been used for pathological grading of malignancies. It is well known that the mode of invasion classified by the YK classification is a potentially important predictor of cervical lymph node metastasis and patient outcomes in patients with oral squamous cell carcinomas [40, 41]. Importantly, by the mode of invasion, the number of LpMab-23-dull/negative cases was significantly more numerous in the YK-1/YK-2/YK-3 group than in the YK-4C and YK-4D groups with high-risk for late cervical lymph node metastasis (Figure 1, 2, Table 1). In the YK-4C and YK-4D group, the number of LpMab-23-positive cases was larger than the number of LpMab-23-dull/negative cases. Further, there were significantly more of LpMab-23-dull/negative cases in patients without late cervical lymph node metastasis than in the cases with metastasis (Figure 1, 2, Table 1). Active lymphangiogenesis was more common among LpMab-23 positive cases with no reaction to D2-40 (Figure 2). These differences suggest that LpMab-23-dull/negative findings are linked to good prognostic factors and long lymphatic metastasis-free interval for tongue cancer patients.

There were also a statistically significantly differences in LpMab-23, as well as in age and mode of invasion for the five-year metastasis-free survival rate (MFS) and the Kaplan–Meier curves (Table 2). The Kaplan–Meier curves of MFS were significantly lower in the patients with LpMab-23-positive than in the patients with LpMab-23 dull/negative (Figure 3). The LpMab-23-dull/negative cases showed the highest MFS in all of the clinical/pathological features tested and particularly, MFS of the cases with LpMab-23 positive and with YK-4C and YK-4D decreased to less than 60% in the first year (Figure 3). These suggests that LpMab-23-positive patients may be considered a high-risk group for late cervical lymph node metastasis, like patients aged 66 years or older and with YK-4C and YK-4D in the mode of invasion. In the Cox proportional hazard regression models the comparison of LpMab-23 dull/negative vs positive test showed the highest hazard ratio and statistical significance as an independent predictor in the multivariate analysis among age, mode of invasion, and LpMab-23 reactivity, as well as in the univariate analysis among all of the all clinical and pathological characteristics tested (Table 3). These results suggest that LpMab-23-positive may be considered a very useful predictor of poor prognosis for early stage tongue cancer. A limitation of this study is the small number of patients with early stage tongue cancer a result of the study being retrospective in a single institution. Despite this, we still believe that it would be useful to define the utility of the LpMab-23 antibody in the clinical field soonest possible. Further studies involving advanced tongue cancer would be able to provide more relevant information for establishing evaluation systems of potential cervical lymph nodes.

## PATIENTS AND METHODS

### Subjects

This study was conducted with sixty patients with stage I and II tongue cancer who had undergone partial glossectomy without elective neck dissection for the early stage tongue cancer (aged 33-92 years; median age 66 years). Specimens were taken from tissue obtained at the surgery. The clinical pathology parameters and the follow-up data were obtained from the clinical database, chart review, and an interview. The extent of the tumor and the histopathological grading were classified according to the seventh edition of the UICC/TNM Classification [42]. The mode of invasion was determined according to the Yamamoto-Kohama (YK) classification for oral squamous cell carcinomas with the assessment of the invasion at the tumor host interface corresponding to an infiltrative growth pattern (INF) of digestive tract cancer [43, 44]. The study was approved by the institutional review board of Sapporo Medical University School of Medicine, with written informed consent obtained from all patients.

### Immunostaining

The specimens were 2-μm thick paraffin-embedded sections cut using a rotary microtome, mounted on 3-aminopropyltriethoxysilane-coated slides, deparaffinized in changes of xylene, rehydrated in decreasing concentrations of ethanol, and rinsed with deionized distilled water for 10 min. The slides were immersed in 10 mM sodium citrate buffer (pH 6.0) and boiled in a microwave oven for 10 min to break formalin-formed protein cross-links masking the PDPN antigen. Immunohistochemistry was performed using the avidin-biotin peroxidase complex technique. After the boiling, the sections were rinsed by 10 mM PBS and then immersed for 10 minutes in methanol containing 3% hydrogen peroxide, followed by incubation in 0.1% goat serum for 30 minutes at room temperature. The sections were then incubated with a 1 μg/ml D2-40 (Agilent Technologies Inc., Santa Clara, CA, USA) and 20 μg/ml LpMab-23 which has been established as a cancer specific mAb [26] diluted into the blocking solution at 4°C overnight followed by signal development processes using the EnVision Detection System (Agilent Technologies, Inc., Santa Clara, CA, USA). The sections were counterstained with Mayer's hematoxylin (Agilent Technologies). All procedures were performed after washing with 10 mM PBS for 30 min at room temperature. After the treatment with primary antibodies the sections were washed three times in PBS for 10 min, immunostained for 0.5 hr at 20°C with 0.1 μg/ml of peroxidase-conjugated goat anti-mouse and goat anti-rat IgGs (Agilent Technologies), and visualized with a DAB peroxide substrate kit (Agilent Technologies). Expression of PDPN in lymphatic endothelial cells in the stroma served as an internal positive control. The immunostained sections were mounted in 50% polyvinylpyrrolidone solution and examined by microscopy. The immunoreactivity of D2-40 and LpMab-23 to the cancer cells were categorized into two groups: clearly positive and dull (seems to be weakly or false-positive)/negative. The assessment was performed by all co-authors.

### Statistical analysis

The correlation between the immunoreactivity of LpMab-23 and the clinical/pathological parameters were analyzed using the Pearson Chi-square test. Clinical outcomes (metastasis-free survival; MFS) were assessed by the Kaplan–Meier method and the differences between the survival curves were analyzed using the log-rank test. To evaluate the correlations between the survival rate and clinical/pathological parameters, univariate and multivariate regression analyses according to the Cox proportional hazards regression models were used, and the hazard ratios with 95% CI and P-values were determined. All tests were two-sided, and P-values below 0.05 were considered statistically significant. Four co-authors evaluated samples, independently. Ratios were rounded off to the 1st (Table 1, 2) or 2nd decimal places (Table 3).
